# Capsaicin Potentiates Anticancer Drug Efficacy Through Autophagy-Mediated Ribophorin II Downregulation and Necroptosis in Oral Squamous Cell Carcinoma Cells

**DOI:** 10.3389/fphar.2021.676813

**Published:** 2021-08-27

**Authors:** Yi-Ching Huang, Tien-Ming Yuan, Bang-Hung Liu, Kai-Li Liu, Chiung-Hua Wung, Show-Mei Chuang

**Affiliations:** ^1^ Institute of Biomedical Sciences, National Chung Hsing University, Taichung, Taiwan; ^2^ Department of Surgery, Feng Yuan Hospital, Ministry of Health and Welfare, Taichung, Taiwan; ^3^ Department of Nutrition, Chung Shan Medical University, Taichung, Taiwan; ^4^ Department of Nutrition, Chung Shan Medical University Hospital, Taichung, Taiwan; ^5^ Biotechnology Center, National Chung Hsing University, Taichung, Taiwan

**Keywords:** capsaicin, ribophorin II, autophagy, apoptosis, necroptosis, DNA damage

## Abstract

The ability of capsaicin co-treatment to sensitize cancer cells to anticancer drugs has been widely documented, but the detailed underlying mechanisms remain unknown. In addition, the role of ribophorin II turnover on chemosensitization is still uncertain. Here, we investigated capsaicin-induced sensitization to chemotherapeutic agents in the human oral squamous carcinoma cell lines, HSC-3 and SAS. We found that capsaicin (200 μM) did not induce remarkable apoptotic cell death in these cell lines; instead, it significantly enhanced autophagy with a concomitant decrease of ribophorin II protein. This capsaicin-induced decrease in ribophorin II was intensified by the autophagy inducer, rapamycin, but attenuated by the autophagy inhibitors, ULK1 inhibitor and chloroquine, indicating that the autophagic process was responsible for the capsaicin-induced down-regulation of ribophorin II. Co-administration of capsaicin with conventional anticancer agents did, indeed, sensitize the cancer cells to these agents. In co-treated cells, the induction of apoptosis was significantly reduced and the levels of the necroptosis markers, phospho-MLKL and phospho-RIP3, were increased relative to the levels seen in capsaicin treatment alone. The levels of DNA damage response markers were also diminished by co-treatment. Collectively, our results reveal a novel mechanism by which capsaicin sensitizes oral cancer cells to anticancer drugs through the up-regulation of autophagy and down-regulation of ribophorin II, and further indicate that the induction of necroptosis is a critical factor in the capsaicin-mediated chemosensitization of oral squamous carcinoma cells to conventional anticancer drugs.

## Introduction

Certain phytochemicals have exhibited great potential to be anticancer agents, enhance the sensitivity of cancer cells to anticancer drugs, or act as cancer-preventing agents, due to their low intrinsic toxicity in normal cells but prominent effects in cancerous cells. Capsaicin, which is the principal pungent alkaloid of red chili, exhibits a wide variety of biological effects and has been the target of extensive research (Rumsfield and West, 1991). Recently, the anticancer and chemopreventive properties of capsaicin have received increasing research interest (Clark and Lee, 2016; Patowary et al., 2017). Capsaicin has been shown to interfere with multiple mechanisms that are central to cancer progression, such as by triggering apoptosis, cell cycle arrest (Clark and Lee, 2016), and/or autophagy (Oh et al., 2008). Mechanistically, capsaicin treatment has been reported to enhance reactive oxygen species (ROS) generation, inhibit activation of the NF-kB, STAT3, MAPK, PI3K-AKT, hedgehog and β-catenin signaling pathways, and increase the activation of ASK1 in a variety of cancer cells (Ranjan et al., 2015).

The ability of capsaicin to restore the sensitivity of cancer cells to conventional chemotherapeutic drugs has attracted considerable attention. The combination of capsaicin plus various anticancer drugs, including 5-fluorouracil (5-FU), pirarubicin, camptothecin, gemcitabine, sorafenib, doxorubicin, and cisplatin, has exhibited synergistic effects against different cancer cells *in vitro* and *in vivo* (Hong et al., 2015; Zheng et al., 2016; Friedman et al., 2017; Vendrely et al., 2017; Dai et al., 2018; Li et al., 2018; Wang et al., 2018). For example, subtoxic concentrations of capsaicin synergistically potentiate the anticancer activity of cisplatin in human osteosarcoma cells *in vitro* and in a xenograft model (Wang et al., 2018). Combination treatment with capsaicin and resveratrol synergizes gemcitabine to reduce tumor growth and rescues the full efficiency of low-dose gemcitabine (the main first-line therapy for pancreatic cancer) in a pancreatic cancer model (Vendrely et al., 2017). Importantly, the addition of capsaicin to a reduced dose of gemcitabine achieves the same efficiency as mono-treatment with a complete dose. Thus, capsaicin could rescue the efficiency of anticancer drugs in patients displaying adverse anticancer drug-related side effects, leading clinicians to revise the treatment dose. Capsaicin also reportedly synergizes with camptothecin to increase apoptosis in human small cell lung cancers (Friedman et al., 2017). Taken together, the previous reports indicate that phytochemicals, such as capsaicin, could be beneficial food components that may serve as chemotherapeutic adjuvants for the treatment of human cancers. However, the mechanisms underlying capsaicin-mediated drug sensitization are poorly understood.

N-linked glycosylation is a protein modification that is critical for glycoprotein folding, stability, and cellular localization, and thereby plays crucial roles in various biological processes, including cell adhesion, proliferation, and cellular signaling. This essential reaction is catalyzed by the N-oligosaccharyl transferase complex, which is a highly conserved multisubunit complex located exclusively in the membranes of the rough endoplasmic reticulum (ER) (Kelleher et al., 1992). Ribophorin II is an essential subunit of the N-oligosaccharyl transferase (OST) complex that conjugates high mannose oligosaccharides to asparagine residues in the N-X-S/T consensus motif of nascent polypeptide chains (Kelleher et al., 1992). The most well-known substrate of ribophorin II is P-glycoprotein, which mediates multidrug resistance (Honma et al., 2008). In addition to its role in N-linked glycosylation, ribophorin II is an important molecular marker in various cancers and has been associated with drug resistance in solid cancers (Honma et al., 2008; Kurashige et al., 2012; Fujita et al., 2015; Yuan et al., 2015). Honma et al. established that glycosylation of P-glycoprotein 1 (encoded by the MDR1 gene, official gene symbol ABCB1) is regulated by ribophorin II (Honma et al., 2008). Silencing of ribophorin II reportedly facilitates the docetaxel-dependent apoptosis and cell growth inhibition of human breast cancer cells by reducing the N-linked glycosylation and membrane localization of P-glycoprotein. Furthermore, the *in vivo* delivery of ribophorin II siRNA inhibits tumor growth in mice given docetaxel (Honma et al., 2008). Increasing evidence indicates that ribophorin II is also multifunctional, in that it tightly regulates tumor initiation and malignancy. Downregulation of ribophorin II has been reported to inhibit cancer cell proliferation and metastasis in osteosarcoma (Fujiwara et al., 2014), lung cancer (Fujita et al., 2013; Fujita et al., 2015), and breast cancer (Takahashi et al., 2013; Tominaga et al., 2014). Integrated transcriptional profiling and genomic analyses reveal that ribophorin II is a promising biomarker in colorectal cancer (Zhang et al., 2015) and promotes colorectal cancer proliferation via regulating the glycosylation status of EGFR (Li et al., 2017). Higher ribophorin II expression is associated with poor patient prognosis, suggesting that ribophorin II expression could be monitored as a new prognostic factor in human tumors.

Chemotherapy is an effective strategy to treat human tumors, but the extent of this efficacy is limited. The toxicity and acquisition of intrinsic resistance after long-term application of chemotherapeutic agents remain major obstacles. Increases in drug efflux are often responsible for enhanced drug resistance and are frequently due to increased expression of ABC (ATP-binding cassette) transporter proteins. Multidrug resistance, whereby tumors exhibit resistance to chemically unrelated anticancer drugs, is the one of the most formidable challenges in the field of cancer chemotherapy. P-glycoprotein is the first mediator of multidrug resistance to be characterized at the molecular level, and its localization at the cell membrane has been shown to be important to the drug resistance of cancer cells. The targeting of P-glycoprotein by small molecule compounds or antibodies has proven to be an effective strategy for overcoming multidrug resistance in cancer (Tsuruo et al., 2003). Nabekura et al. reported that capsaicin has an inhibitory effect on P-glycoprotein, leading to accumulation of daunorubicin (Nabekura et al., 2005) through a mechanism that is not yet understood in detail. Honma et al. reported that silencing of ribophorin II reduces the glycosylation and membrane localization of P-glycoprotein, thereby sensitizing cancer cells to docetaxel (Honma et al., 2008).

The co-application of chemotherapeutic drugs with natural compounds can improve the efficacy of anticancer therapy. In the last decade, it has been widely accepted that capsaicin exhibits anticancer activity. Most of the studies exploring the anticancer activity of capsaicin have focused on the mechanisms underlying capsaicin-induced apoptosis, cell cycle arrest, and signaling pathway modulation. Oral squamous cell carcinoma is the most common oral cancer with more than 90% of the histological classification in men. Capsaicin is excellently absorbed by oral mucosa. In this study, we further demonstrate the cancer-specific chemosensitizing effect of capsaicin for four paradigmatic anticancer drugs (5-FU, cisplatin, docetaxel, and doxorubicin) and investigate the underlying mechanisms of cytotoxicity in two oral squamous carcinoma cell lines. Our data show for the first time that capsaicin induces autophagy to enhance the turnover of ribophorin II. When applied in combination with the tested chemotherapeutic agents, capsaicin increases their cytotoxicity, seemingly by abolishing the DNA-damage response and promoting a switch from apoptosis to necroptosis, thereby diminishing the cell proliferation of oral squamous carcinoma cells.

## Materials and Methods

### Cell Culture

The HSC-3 cell line, representing a human squamous cell carcinoma of the tongue with high metastatic potential, and the SAS cell line, representing a poorly differentiated human squamous cell carcinoma of the tongue, were purchased from the Japanese Collection of Research Bioresources Cell Bank (Osaka, Japan). Fetal bovine serum (FBS) and penicillin/streptomycin were obtained from Invitrogen (Carlsbad, CA, United States). All cells were cultured in DMEM (Invitrogen) supplemented with 10% FBS, at 37 °C in a humidified incubator containing 5% CO_2_ in air.

### Chemicals and Antibodies

Capsaicin, chloroquine, rapamycin, and the ULK1 inhibitor (SBI-0206965) were obtained from Sigma-Aldrich (St. Louis, MO, United States). MG132 was purchased from TOCRIS Bioscience (Ellisville, MO, United States). Specific antibodies against ribophorin II and β-actin were obtained from Santa Cruz Biotechnology (Dallas, TX, United States). Antibodies against poly (ADPribose) polymerase, caspase-3, phospho-elF2α, XBP1, ATG5, LC3B, phospho-MLKL (S358), and phospho-RIP3 (S227) were obtained from Cell Signaling Technology (Beverly, MA, United States). The antibody against p62 was purchased from Sigma-Aldrich. The antibody against phospho-IRE1α was obtained from Abcam (Cambridge, United Kingdom). The antibody against TRIB3 was obtained from Atlas Antibodies (Bromma, Sweden). The antibody against beclin 1 was purchased from Novus Biologicals (Centennial, CO, United States). The antibody against phospho-MLKL (T357/S358/S360) was purchased from ABclonal (Woburn, MA, United States).

### Western Blot Analysis

Cell extracts were prepared in a lysis buffer consisting of 20 mM Tris-HCl (pH 7.4), 100 mM NaCl, 5 mM EDTA, and a protease inhibitor cocktail (Roche, Germany). Volumes of extract containing equal amounts of proteins were separated by sodium dodecyl sulfate polyacrylamide gel electrophoresis (SDS-PAGE). The proteins were transferred onto polyvinylidene difluoride (PVDF) membranes (Millipore, Bedford, MA, United States), and the membranes were blocked, washed, and probed with primary antibodies. Each primary antibody was removed by washing, and the membranes were incubated with an appropriate horseradish peroxidase-conjugated goat anti-mouse or anti-rabbit secondary antibody (Jackson Immuno Research Laboratories, West Grove, PA, United States) for 1 h. The blots were washed again, and then developed using enhanced chemiluminescence (ECL) reagents, according to the manufacturer’s instructions (Millipore). The images were quantified by ImageJ software (Schneider et al., 2012) to obtain the relative protein levels. The images shown are representative of at least three independent experiments carried out under the same conditions.

### Reverse Transcription Polymerase Chain Reaction Analysis

RNA was isolated from the cultured cells using the TRIzol reagent (Invitrogen) according to the manufacturer’s instructions. cDNA was synthesized from 2 μg of total RNA by reverse transcription, using an ImProm-II Reverse Transcriptase kit (Promega, Madison, WI, United States) and oligo (dT) 12–18 primers. The resulting cDNA was used for the subsequent PCR assays. Ribophorin II was amplified with specific primers (forward, 5′GCC​AGG​AAG​TGG​TGT​TTG​TT3′ and reverse, 5′ACA​GAG​CGA​AGA​GCA​GAA​GC3′) and a thermal cycling program consisting of 30 cycles of 95°C for 1 min, 55°C for 1 min, and 72°C for 1 min β-Actin was amplified as an internal control using specific primers (forward, 5′AGA​GCT​ACG​AGC​TGC​CTG​AC3′ and reverse, 5′CAC​CTT​CAC​CGT​TCC​AGT​TT3′).

### Cell Counting Kit-8 Assay

Cell viability was determined using a Cell Counting Kit-8 (CCK-8) (Dojindo Molecular Technologies, Kumamoto, Japan) according to the manufacturer’s instructions. Briefly, cells (5 × 10^3^/well) were seeded to 96-well plates, incubated overnight, and treated with capsaicin or combination of capsaicin with conventional anticancer drugs. After 48 h, 10 μl of CCK-8 reagent was added to each well at 37°C, as recommended by the manufacturer. Finally, the spectrophotometric absorbance was measured using a microplate reader (Multiskan FC; Thermo Fisher Scientific, Vantaa, Finland) at 450 nm. The data were obtained from five or six experiments, and each experiment was performed in triplicate.

### xCELLigence Real Time Cell Analysis

Cell proliferation was assayed by an xCELLigence Real Time Cell Analysis System (RTCA) (ACEA Biosciences, San Diego, CA, United States). Briefly, cells (5 × 10^3^/well) were suspended in medium containing 10% FBS, seeded in E-plates, and placed onto the RTCA station. Cell proliferation was monitored by measuring cell impedance, and the normalized cell index was determined as previously described (Lin et al., 2018). Data acquisition and analysis were performed with the RTCA software (version 2.0) as described previously (Liang et al., 2020).

### Immunofluorescence Staining

Cells grown on cover slides were washed in phosphate buffered saline (PBS), fixed in 4% formaldehyde/PBS, washed in PBS, and blocked in 5% bovine serum albumin/PBS. The cells were incubated with primary antibodies (in 5% bovine serum albumin/PBS) overnight at 4°C and washed. Binding of primary antibodies was detected with rhodamine-conjugated goat anti-rabbit (Millipore). Fluorescence images were obtained using an Olympus IX71 fluorescence microscope (Olympus, Tokyo, Japan). The images shown are representative of at least three independent experiments carried out under the same conditions.

### Flow Cytometry

Cells cultured in 6-cm dishes were trypsinized and collected by centrifugation. Each cell pellet was washed once with PBS and fixed overnight in 70% ethanol. The fixed cells were washed twice with PBS, stained with 50 μg/ml propidium iodide (PI; Sigma-Aldrich) and 200 μg/ml RNaseA (Sigma-Aldrich) in PBS for 30 min, and analyzed for their cell cycle distribution using a Beckman Coulter FC500 (Beckman Coulter Inc. Brea, CA, United States). For detection of acidic vesicles in cells, each cell pellet was washed and stained with acridine orange (0.1 μg/ml; Sigma-Aldrich) for 15 min at 37°C before analysis. The images shown are representative of at least three independent experiments carried out under the same conditions.

### Proximity Ligation Assay

For proximity ligation assay (PLA), cells were grown overnight on 18-mm coverslips, fixed, permeabilized, and blocked as recommended by the manufacturer (DuoLink *In Situ* Fluorescence kit, Sigma-Aldrich). Cells then were incubated with mouse anti-ribophorin II and rabbit anti-LC3B (both diluted 1:200) overnight at 4°C. The cells were washed, treated for 1 h with the DuoLink *in situ* PLA probes, anti-mouse MINUS, and anti-rabbit PLUS, washed again, and incubated with ligase-containing ligation buffer for 30 min at 37°C. For amplification, the reactions were treated with polymerase in amplification buffer and incubated for 100 min at 37°C. The cells were washed, incubated with DuoLink *in situ* mounting medium containing DAPI (to stain nuclei) for 15 min, and subjected to analysis. The images of *in situ* PLA were viewed and captured using an IX71 fluorescence microscope. The same analytic parameters were used consistently throughout all experiments. The images shown are representative of at least three independent experiments carried out under the same conditions.

### Measurement of Cellular Accumulation of Rhodamine 123

The accumulation of rhodamine 123 (Sigma-Aldrich), a fluorescent substrate of P-glycoprotein, in cells was measured using a flow cytometer (Beckman Coulter) equipped with an ultraviolet argon laser (excitation at 488 nm and emission at 530 ± 15 nm), as described previously (Nabekura et al., 2005). Cells were incubated with 0.1 μM rhodamine 123 in the absence or presence of 200 μM capsaicin for 30 min at 37°C. The cells were then washed with ice-cold PBS and trypsinized, and the fluorescence intensity of rhodamine 123 in individual cells was measured immediately by a flow cytometer. The images shown are representative of at least three independent experiments carried out under the same conditions.

### Detection of Lysosomes

For lysosome staining, cells were treated with capsaicin for the indicated times and then stained with LysoTracker Red DND-99 (Thermo Fisher Scientific) in serum-free medium for 30 min at 37°C. The cells were then washed twice with PBS, and the fluorescence intensity was detected immediately by a flow cytometer. The images shown are representative of at least three independent experiments carried out under the same conditions.

### Detection of Autophagy

The DAPGreen reagent (Dojindo Molecular Technologies) was used to measure the formation of autophagosomes, as directed by the manufacturer. Briefly, cells were cultured overnight, the culture medium was discarded, and the cells were washed with fresh culture medium. The cells were incubated with DAPGreen working solution at 37°C for 30 min, the solution was discarded, and the cells were washed twice with culture medium and incubated with capsaicin-containing culture medium at 37°C for the indicated times. The formation of autophagosomes was observed under an Olympus IX71 fluorescence microscope. The images shown are representative of at least three independent experiments carried out under the same conditions.

### Combined Drug Analysis

The combined effect of capsaicin and anticancer drugs on human oral squamous carcinoma cells was evaluated by CompuSyn software (ComboSyn, Inc. Paramus, NJ, United States) using a combination index (CI)-isobologram equation that allows quantitative determination of drug interactions, as described previously (Chou, 2006; 2010). The combined effect was classified as follows: CI < 1 implied synergism, CI = 1 additive, and CI > 1 implied antagonism.

### Statistical Analysis

For statistical analysis, each experimental value was compared to its corresponding control. The statistical significance of differences between mean values was estimated using the *t*-test. *p < 0.05* was considered statistically significant. Data are expressed as the mean ± SD for the indicated number of biological replicates.

## Results

### Capsaicin Inhibits Cell Proliferation but Does Not Remarkably Induce Apoptosis in Oral Squamous Carcinoma Cells

**FIGURE 1:**
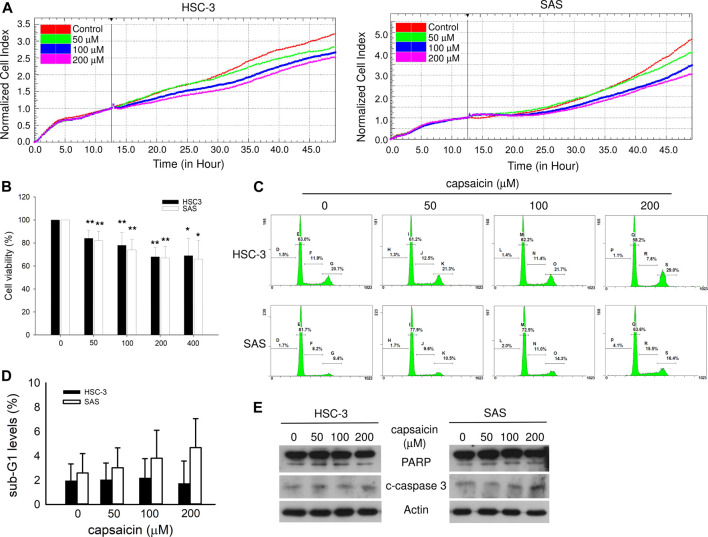
Effect of capsaicin on oral cancer cell growth and apoptosis. **(A)** The cell proliferation of HSC-3 and SAS cells treated with different concentrations of capsaicin, as measured by RTCA. **(B)** Cell viability after capsaicin treatment for 48 h was assayed by Cell Counting Kit-8 (CCK-8). **(C,D)** The levels of apoptosis after capsaicin treatment for 48 h were analyzed by flow cytometry, and apoptosis-related proteins were detected by Western blot analysis **(E)**. Data are representative of three to six independent experiments.

HSC-3 is isolated from a highly metastatic cell line to lymph node in human oral squamous cell carcinoma by orthotopic implantation in nude mice. SAS is established from poorly differentiated human squamous cell carcinoma of the tongue. These two human oral squamous carcinoma cell lines are suitable model for the study of squamous cell carcinoma on cell cytotoxicity, angiogenesis, and metastasis *in vitro* and *in vivo*. Firstly, to assess the capsaicin sensitivity of human oral tumor cell lines, we treated HSC-3 and SAS cells with increasing doses of capsaicin and determined cell proliferation. RTCA showed that the cell proliferation rate was slightly attenuated by increasing concentrations of capsaicin (Figure 1A). CCK8 assays showed that 200 μM capsaicin decreased cell viability to 75% of control groups in both cell lines (Figure 1B). Analysis of apoptosis markers confirmed that capsaicin slightly and dose-dependently increased the levels of sub-G1 cells, cleaved PARP, and active caspase-3. However, the capsaicin-induced increases in apoptotic cell death were not significant in HSC-3 or SAS cells relative to controls; in HSC-3 cells, in particular, the difference was unobvious (Figures 1C–E). These data suggested that capsaicin did not induce remarkable apoptotic cell death at concentrations of 200 μM or higher.

### Capsaicin Enhances ER Stress and Autophagy

**FIGURE 2:**
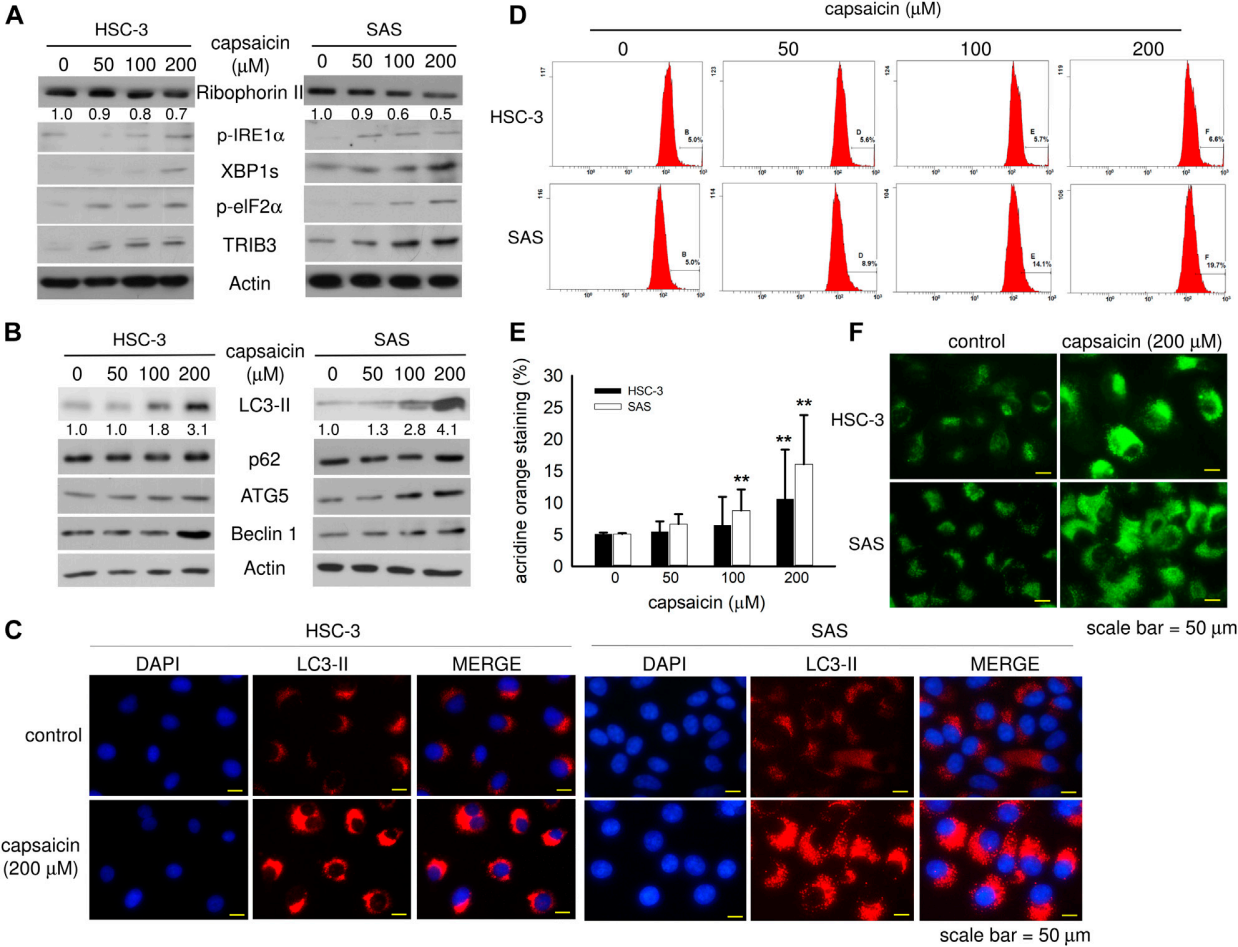
Capsaicin dose-dependently down-regulates ribophorin II and enhances autophagy in HSC-3 and SAS cells. **(A,B)** Cells were treated with different concentrations of capsaicin for 48 h and the levels of ER stress- and autophagy-related proteins were measured by Western blot analysis. **(C)** Cells were treated with or without 200 μM capsaicin for 48 h followed by immunofluorescence staining to detect the levels of LC3-II. **(D,E)** Cells were treated with different concentrations of capsaicin for 48 h. The cells were then collected in microcentrifuge tubes and treated with 0.1 μg/ml acridine orange for 30 min before being analyzed by flow cytometry. **(F)** After treatment with capsaicin, cells were stained with the DAPGreen reagent, as described in the Materials and Methods. Data are representative of five to ten independent experiments. Scale bar = 50 μm.

The expression level of ribophorin II has been proposed as a predictive marker for drug resistance, cell proliferation, and motility in various cancers. However, little is known about the correlation between ribophorin II expression and the response of oral squamous carcinoma cells to capsaicin, a natural compound with chemopreventive effect in a variety of cancers (Clark and Lee, 2016). Interestingly, we found that capsaicin concentration-dependently decreased the level of ribophorin II, and that this was accompanied by up-regulation of ER stress markers, such as phospho-IRE1α, spliced XBP1, phospho-eIF2α, and TRIB3 (Figure 2A). Capsaicin enhanced autophagy in both cell lines as reflected by increased protein levels of LC3-II, p62, ATG5, and beclin1 (Figure 2B). Immunofluorescence imaging of the expression and subcellular distribution of LC3-II revealed that the LC3-II staining was more intense in cells treated with 200 μM capsaicin compared with control cells (Figure 2C). Acridine orange staining of acidic vacuoles revealed that capsaicin increased the formation of autolysosomes (Figures 2D,E). The DAPGreen signal, a small fluorescent molecule that stains autophagosomes, was enhanced by capsaicin, indicating an increase of autophagy (Figure 2F). This increase in autophagy was greater in SAS cells than that in HSC-3 cells, although it was significant in both cases. Together, our data suggest that capsaicin might enhance autophagy through ER stress, and that this might down-regulate ribophorin II expression.

### Capsaicin Down-Regulates Ribophorin II Protein Through Autophagy

**FIGURE 3:**
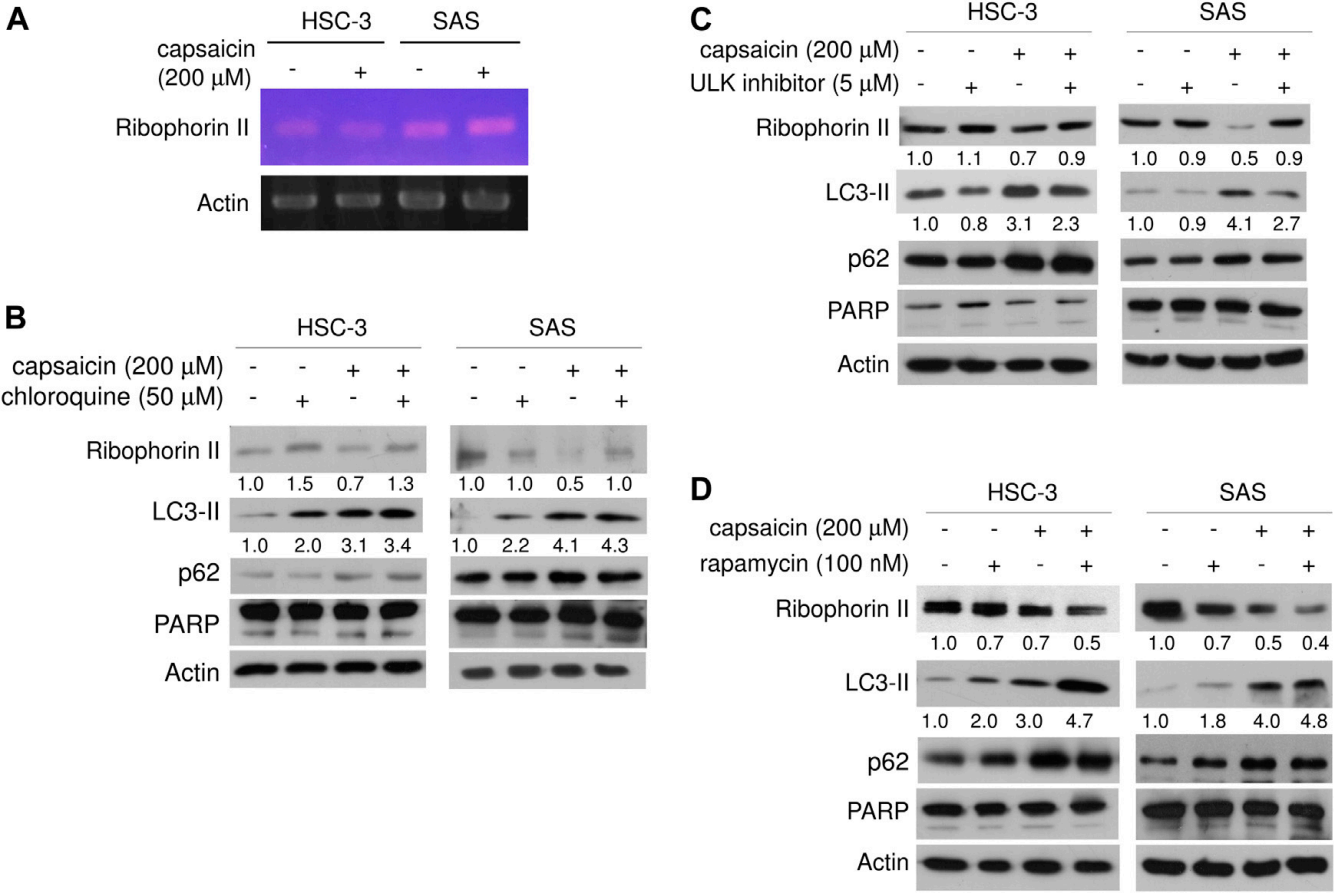
Capsaicin induces autophagy, which down-regulates ribophorin II. **(A)** After capsaicin treatment for 24 h, mRNA levels of ribophorin II in HSC-3 and SAS cells were assayed by RT-PCR. **(B)** Cells were pre-treated with capsaicin for 42 h, co-treated with chloroquine for another 6 h, and then Western blot assays were used to examine the levels of ribophorin II, LC3-II, p62, and cleaved PARP. **(C)** Cells were pre-treated with capsaicin for 42 h, co-treated with ULK1 inhibitor for another 6 h, and then evaluated with Western blot assays. **(D)** Cells were pre-treated with capsaicin for 24 h, co-treated with rapamycin for another 24 h, and then evaluated with Western blot assays. Data are representative of five to six independent experiments.

**FIGURE 4:**
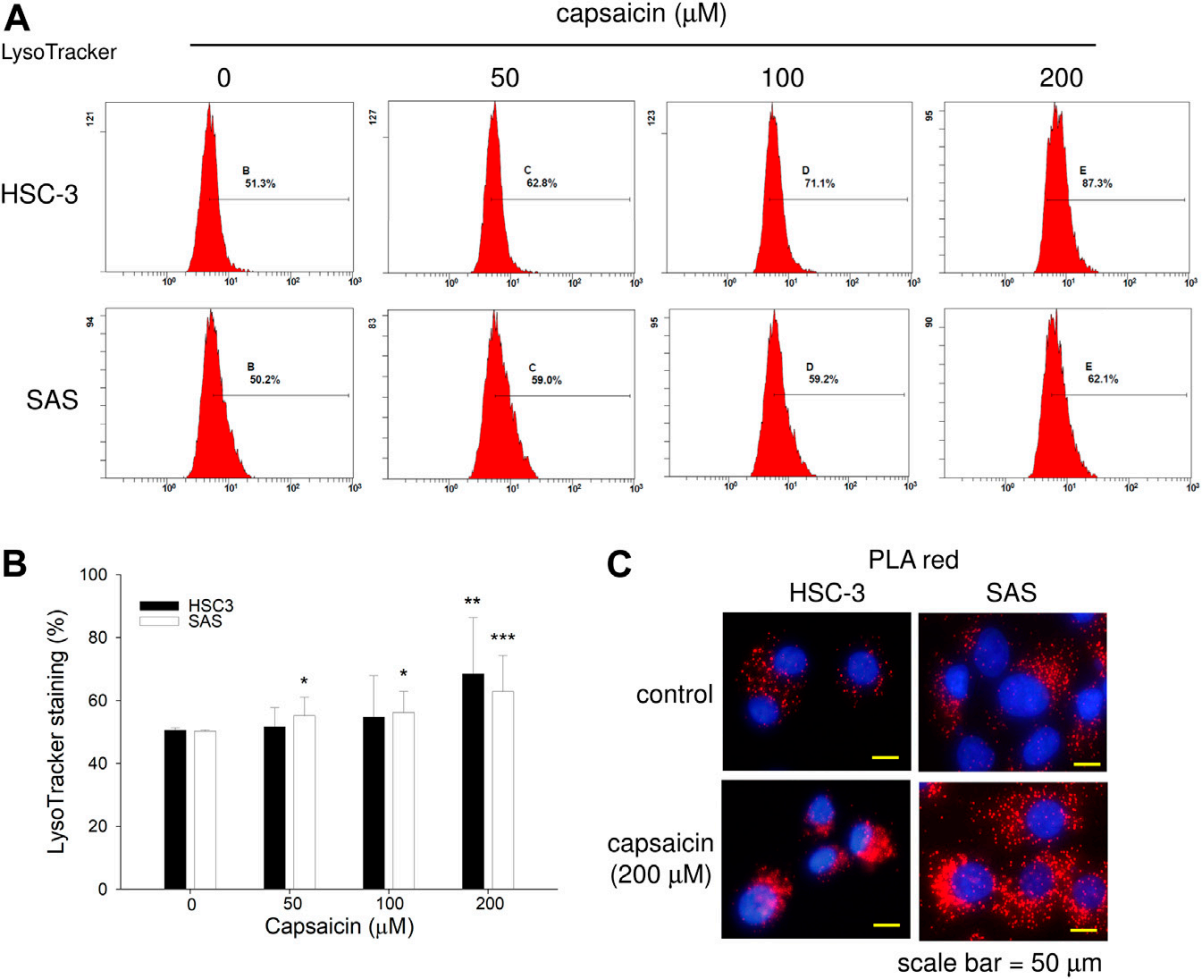
Effect of capsaicin on lysosome function. **(A,B)** Cells were treated with different concentrations of capsaicin for 48 h, treated with 1 μM LysoTracker for 30 min, and analyzed by flow cytometry. **(C)** Cells were treated with capsaicin for 24 h, and PLA was used to examine the co-localization of LC3-II and ribophorin II. Data are representative of three to six independent experiments. Scale bar = 50 μm.

We next explored the mechanism through which capsaicin down-regulated ribophorin II in HSC-3 and SAS cells. RT-PCR showed that capsaicin did not attenuate the mRNA expression level of ribophorin II (Figure 3A), suggesting that the capsaicin-induced decrease of ribophorin II did not result from a decrease in transcription. MG132 treatment did not significantly reverse the capsaicin-mediated down-regulation of ribophorin II (data not shown), indicating that ubiquitin proteasome system might not play a role in the down-regulation of ribophorin II by capsaicin. Since capsaicin induced autophagy in these cell lines, we postulated that autophagy could contribute to the down-regulation of ribophorin II upon capsaicin treatment. We thus further assessed the autophagy induced by capsaicin and investigated its potential role in the capsaicin-triggered down-regulation of ribophorin II. Autophagosome accumulation, which leads to autophagy, can be caused by the increased formation of autophagosomes or by the inhibition of their fusion with lysosomes. To support this speculation, we examined the effect of chloroquine, which prevents the fusion of autophagosomes with lysosomes, in our system. Our results revealed that chloroquine further enhanced the capsaicin-induced protein level of LC3-II, which is widely used to monitor autophagy, and prevented the capsaicin-induced down-regulation of ribophorin II (Figure 3B). This result indicated that autophagy might be upstream of the down-regulation of ribophorin II under capsaicin treatment. Consistent with this, an inhibitor of ULK-1 (which is key to autophagy initiation) promoted the accumulation of ribophorin II in the presence of capsaicin (Figure 3C). Notably, inhibition of autophagy did not increase the level of cleaved PARP, indicating that capsaicin-induced autophagy may not modulate apoptosis in our system. In contrast, the mTOR signaling inhibitor, rapamycin, efficiently increased the LC3-II protein level and further augmented the capsaicin-mediated decrease of ribophorin II (Figure 3D). Together, these findings suggest that autophagy accounts for the capsaicin-induced down-regulation of ribophorin II in HSC-3 and SAS cells. LysoTracker staining revealed that capsaicin dose-dependently increased the staining intensity of lysosomes in these cell lines (Figures 4A,B). Our *in situ* proximal ligation assay (PLA) additionally demonstrated that ribophorin II co-localized with LC3-II under capsaicin treatment (Figure 4C), indicating that capsaicin-enhanced autophagy is correlated with the down-regulation of ribophorin II.

**FIGURE 5:**
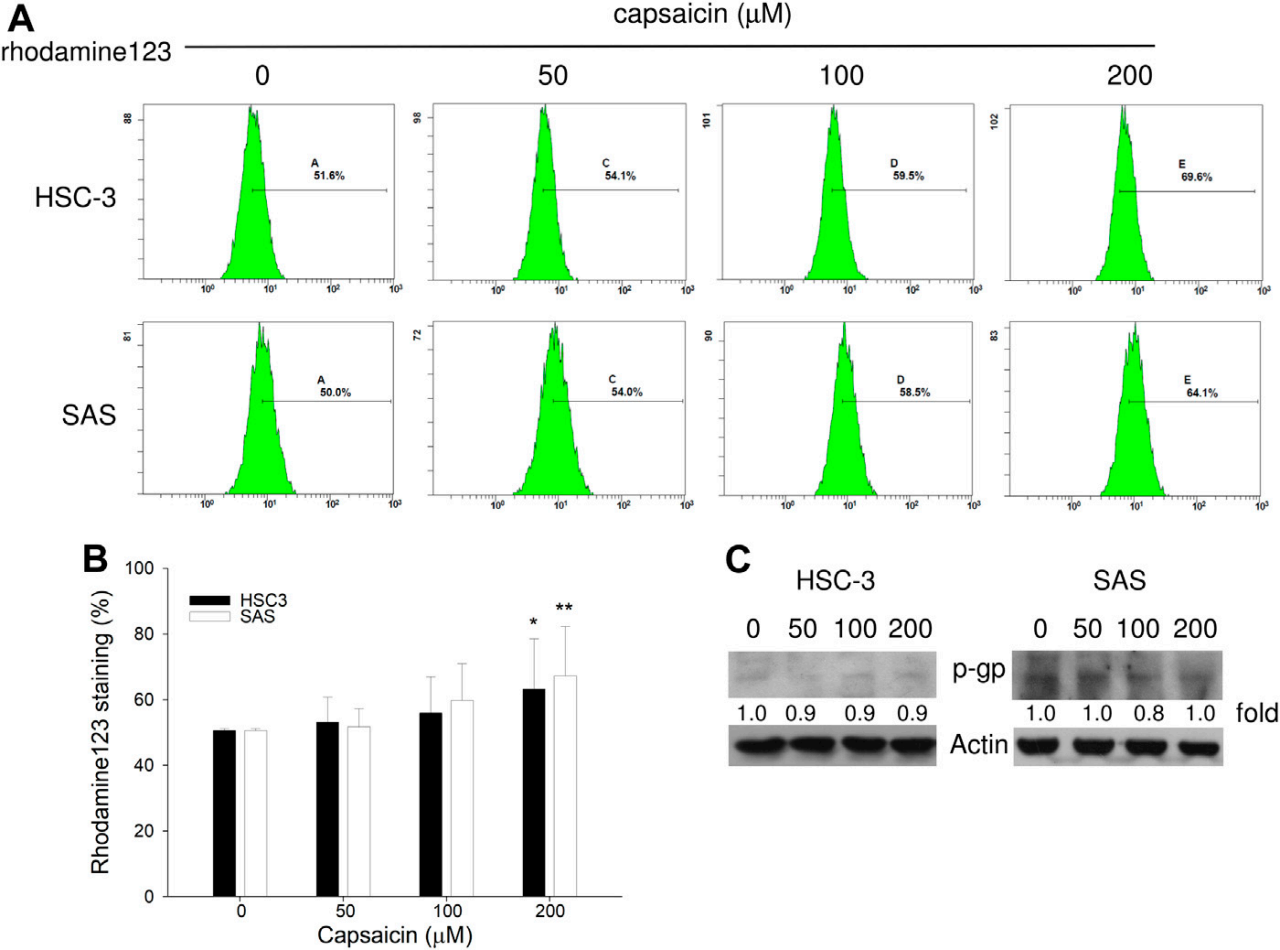
The exclusion of rhodamine 123 is decreased by capsaicin. **(A,B)** Cells were treated with different concentrations of capsaicin for 48 h, treated with 0.1 μM rhodamine 123 for 30 min, and analyzed by flow cytometry. **(C)** Cells were treated with different concentrations of capsaicin for 48 h, and Western blot assays were used to examine the levels of P-glycoprotein (p-gp). Data are representative of three independent experiments.

The most well-known substrate of ribophorin II is P-glycoprotein, which critically mediates multidrug resistance (Honma et al., 2008). Silencing of ribophorin II reduces the N-linked glycosylation and membrane localization of P-glycoprotein, and *in vivo* delivery of ribophorin II siRNA inhibited tumor growth in mice given docetaxel (Honma et al., 2008). To study the impact of capsaicin-mediated autophagy on the function of ribophorin II, we used flow cytometry to measure the cellular accumulation of rhodamine 123, a fluorescent substrate of P-glycoprotein, in the two oral squamous carcinoma cell lines. As shown in Figures 5A,B, capsaicin dose-dependently increased the cellular accumulation of rhodamine 123. Importantly, this treatment did not alter the protein level of P-glycoprotein (Figure 5C). Taken together, our data suggest that the capsaicin-triggered enhancements of autophagy correlates with the down-regulation of ribophorin II, which decreases P-glycoprotein function to inhibit the P-glycoprotein-mediated efflux of rhodamine 123.

### Synergistic Effects and Necroptosis Are Induced by the Application of Capsaicin Plus Anticancer Drugs

**FIGURE 6:**
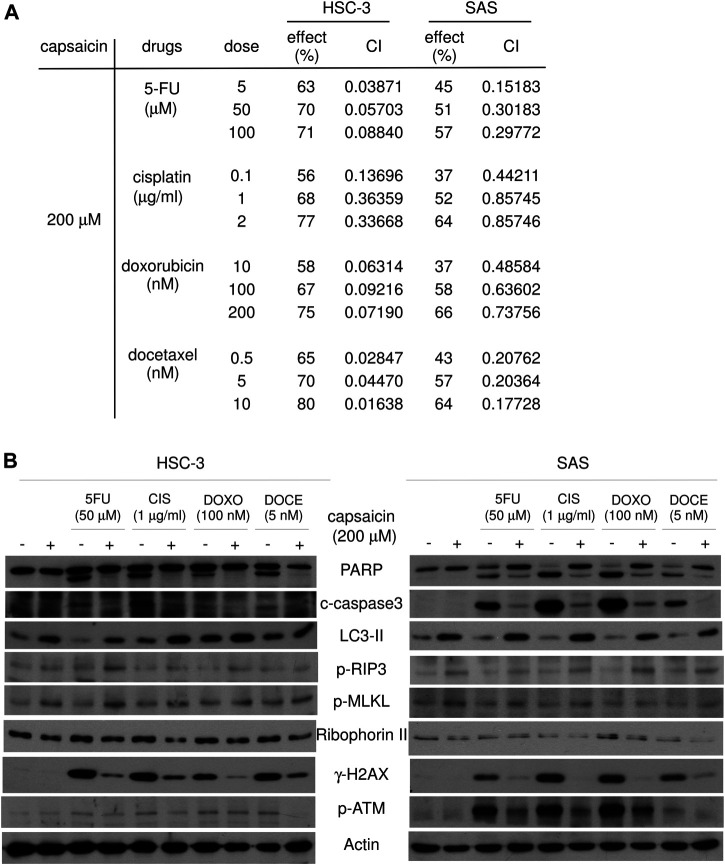
Capsaicin plus each of four anticancer agents show synergistic effects on cell viability and induce necroptosis in HSC-3 and SAS cells. **(A)** Cells were cotreated with 200 μM capsaicin and each of the listed anticancer drugs for 48 h, and cell viability was evaluated by CCK-8. The combined effect of capsaicin and anticancer drugs was evaluated using a combination index (CI)-isobologram equation that allows quantitative determination of drug interactions, as described in the Materials and Methods. **(B)** Cells were co-treated with 200 μM capsaicin and each of the listed anticancer drugs for 48 h, and Western blot assays were used to examine the levels of specific proteins related to apoptosis, autophagy, necroptosis, and the DNA damage responses.

Finally, we investigated whether capsaicin co-treatment could enhance the cytotoxicity of anticancer drugs in oral squamous carcinoma cells. 5-FU, cisplatin, and docetaxel are usually used in the treatment of oral squamous cell carcinoma as the adjuvant radiotherapy following surgery. Doxorubicin is a chemotherapy agent used to treat various cancers in human. The cytotoxic effect of capsaicin in combination with conventional anticancer drugs (5-FU, cisplatin, docetaxel, and doxorubicin) was measured CCK8 assays. The synergistic effect of capsaicin was assessed using the CompuSyn software, which was also used to calculate the combination index (CI). The CI can quantify the synergism or antagonism of two drugs: CI = 1 indicates an additive effect, whereas CI < 1 and CI > 1 indicate synergism and antagonism, respectively. Our CI analysis showed that SAS and HSC-3 cells exhibited potent and synergistic inhibition of cell viability, with CI < 1 for all combinations of 100 and 200 μM capsaicin plus an anticancer drug (Supplementary Figure S1 and Figure 6A). To further investigate the molecular mechanisms underlying the ability of capsaicin to sensitize the tested cell lines to anticancer agent-mediated cytotoxicity, we examined apoptosis. Surprisingly, although the cleavage of PARP, a marker of cells undergoing apoptosis, was increased by the anticancer agents alone, these increases were diminished by 200 μM capsaicin co-treatment (Figure 6B). This suggested that apoptosis might not be the underlying pathway through which oral cancer cells were killed by combined treatment with capsaicin and the tested anticancer agents. Further, the combined treatments slightly increased the LC3-II levels compared to those seen following treatment with each anticancer drug alone (Figure 6B). Recent studies have shown that apoptosis and necroptosis can regulate each other. Interestingly, necroptosis induced by therapeutic agents is often accompanied by autophagy, which may be responsible for suppressing apoptosis and biasing cells toward necroptosis (Gong et al., 2019). At the molecular level, necroptosis is caspase-independent and signals through RIP1, RIP3 and MLKL, the latter of which is the so-called “executioner” when a cell enter necroptotic pathway. MLKL is phosphorylated at Thr357/Ser358, leading to its oligomerization and translocation to the plasma membrane. MLKL oligomers subsequently disrupt membrane integrity and increase the permeability of the membrane (Gong et al., 2019). Here, we found that the levels of phospho-MLKL and phospho-RIP3 were not induced by the four tested anticancer agents; however, they were remarkably increased by capsaicin co-treatment, and thus anti-correlated with the down-regulations of cleaved-PARP and caspase 3 (Figure 6B). Strikingly, capsaicin co-treatment remarkably diminished the chemotherapeutic drug-increased levels of H2AX phosphorylated at serine 139 (γ-H2AX), which is a sensitive molecular marker of DNA damage and repair. One of the earliest events in the DNA damage responses is the recruitment of ATM to the DNA break, and this ATM phosphorylates nearby H2AX at serine 139. The generated γ-H2AX is specifically recognized by DNA repair proteins, leading to their localization at the DNA damage site. Surprisingly, we herein found that the levels of autophosphorylated ATM (at Ser 1981) were diminished by capsaicin co-treatment, compared to the levels seen following the application of each anticancer drug alone (Figure 6B). Based on this, we propose the novel idea that the concomitant use of capsaicin might abolish the DNA damage response of oral cancer cells exposed to chemotherapeutic drugs, which in turn might tip the cellular balance towards cell death. Our findings further suggest that necroptosis, but not apoptosis, might be the major cause for the decrease of cell viability seen following the co-application of capsaicin with the four tested anticancer agents in two oral squamous carcinoma cell lines.

## Discussion

**FIGURE 7:**
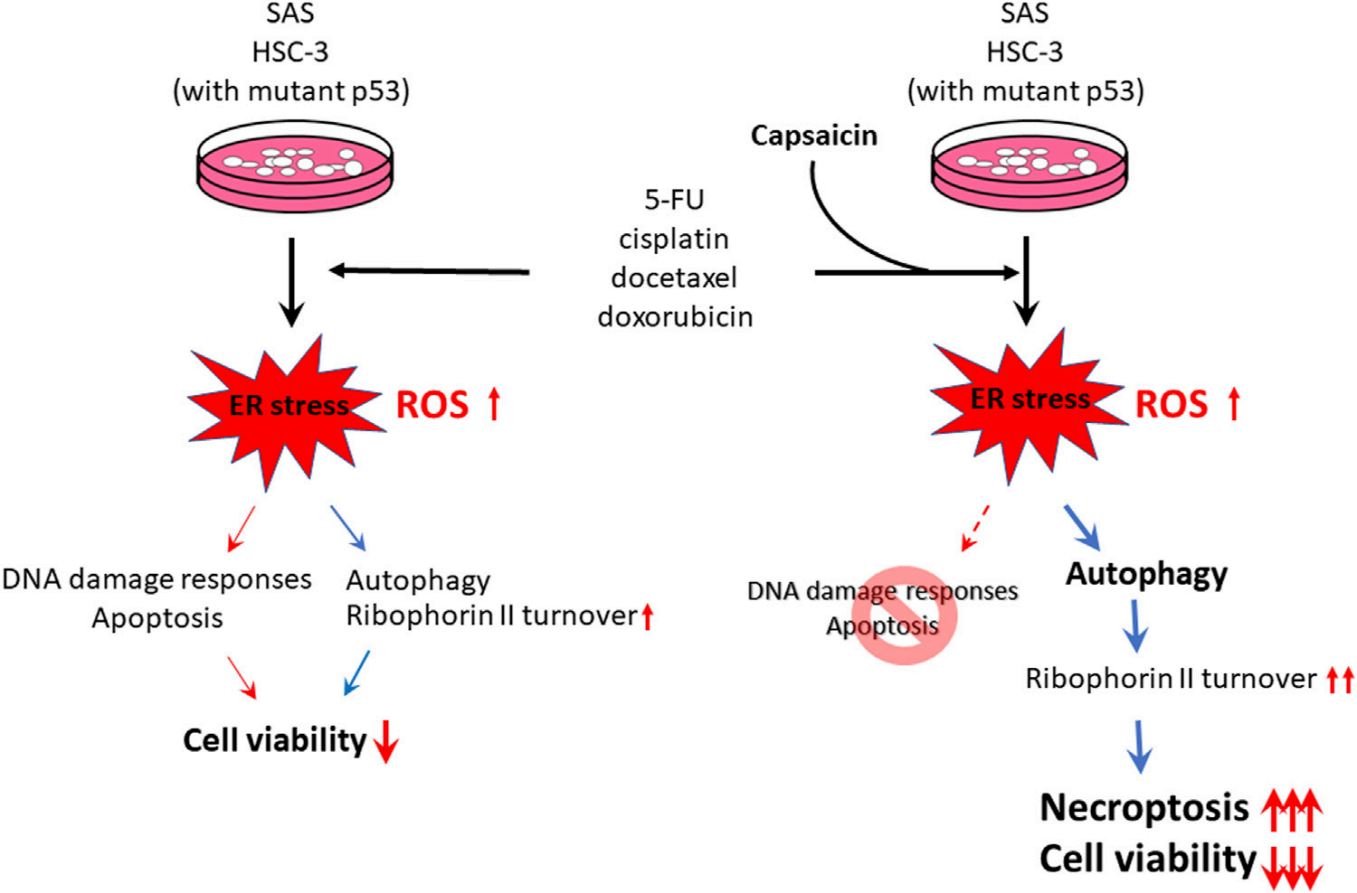
Schematic representation of capsaicin inducing necroptosis when applied in combination with convenient anticancer drugs to HSC-3 and SAS cells. Our present results also suggest that autophagy-enhanced ribophorin II turnover might contribute to capsaicin-mediated cytotoxicity.

Numerous studies have examined the molecular functions and clinical relevance of ribophorin II in a variety of human cancers. Ribophorin II expression status is a predictive marker for drug resistance, cell proliferation, and motility in various cancers, and higher ribophorin II expression is associated with poor patient prognosis (Honma et al., 2008; Kurashige et al., 2012; Fujita et al., 2015; Yuan et al., 2015). Our group previously found that downregulation of ribophorin II alleviates the resistance of gastric cancer cells to multiple chemotherapeutic agents (Yuan et al., 2015). However, the mechanism underlying the turnover of ribophorin II has not been explored. We previously reported that capsaicin, which is highly absorbed by the oral mucosa, suppresses the growth of various cancer cells in culture through inducing apoptosis (Lin et al., 2018). Capsaicin also has been proposed to trigger cell death via autophagy (Oh et al., 2008). Here, we assessed the effects of capsaicin on cell proliferation, apoptosis, autophagy, ER stress, ribophorin II expression, and drug responses in oral squamous carcinoma cells. We found that capsaicin has moderate cytotoxic effects on two oral carcinoma cell lines, and that apoptotic cell death was not provoked by 200 μM capsaicin, as assessed by analysis of the sub-G1 population and the levels of cleaved caspase-3 and PARP. Mechanistically, we found that capsaicin enhanced the turnover of ribophorin II from the ER membrane, likely through autophagy, and that this attenuated ribophorin II-regulated P-glycoprotein function and decreased cell proliferation in human oral squamous carcinoma cells co-treated with anticancer drugs. Interestingly, our results further suggested that capsaicin co-treatment might abolish the DNA damage responses and convert the anticancer agent-mediated cytotoxicity mode from apoptosis to necroptosis (Figure 7). Thus, our results collectively suggest that synergistic co-treatments involving capsaicin could allow the current chemotherapeutic drugs to be applied at lower doses without decreasing their effectiveness, with the added benefit of avoiding and/or overcoming drug resistance.

Various molecular pathways have been investigated for their potential to underlie the synergistic effect of capsaicin on anticancer drugs, such as signaling pathway inhibition, apoptosis activation, and ROS generation. Capsaicin was shown to activate transient receptor potential vanilloid 1 (TRPV1) to prevent the nuclear translocation of proliferating cell nuclear antigen, and was found to inhibit cell growth in a bladder cancer cell line in which TRPV1 is highly expressed (Zheng et al., 2016). Capsaicin also reportedly increases the intracellular accumulation of daunorubicin/vinblastine in KB-C2 cells and that of doxorubicin in Caco-2 cells by inhibiting P-glycoprotein efflux activity, thereby potentiating the anticancer activity of chemotherapeutic agents (Nabekura et al., 2005; Li et al., 2018). These findings indicated that capsaicin may inhibit P-glycoprotein to potentiate the anticancer effects of chemotherapeutics. Here, we further show that capsaicin decreases the ribophorin II protein level by enhancing autophagy; this may inhibit the P-glycoprotein efflux activity to promote the anti-proliferation effects of anticancer agents in human oral squamous carcinoma cells.

Autophagy is a major protein degradation pathway that acts to maintain cellular homeostasis by removing damaged proteins and organelles and recycling normal cellular proteins and organelles. Several chemotherapeutic agents evoke autophagy, which can be cytoprotective or cytotoxic, depending on the cell context, genetic variations, and experimental conditions. The effects of autophagy induced by capsaicin in cancer cells are still controversial. For instance, Lewinska et al. reported that up to 250 μM capsaicin induces genotoxic stress but does not promotes apoptosis in A549 and DU145 cancer cells, and suggested that the higher concentrations of capsaicin might decrease metabolic activity to exert antiproliferative and cytostatic actions (Lewinska et al., 2015). In contrast, capsaicin-induced autophagy was reported to play important roles in the DNA damage responses of breast cancer cells, activating the ATM-DNA-PKcs-PARP-1 axis to protect cells against apoptosis (Yoon et al., 2012). Mutual regulation between autophagy and apoptosis has been reported in capsaicin-treated cancer cells. For example, capsaicin was reported to induce apoptotic cell death in osteosarcoma cells while simultaneously activating the autophagy-ERK signaling axis to counteract a caspase-independent cell death (Chien et al., 2013). Through ROS generation, capsaicin was reported to block autophagy and increase apoptosis among prostate cancer cells, supporting an anticancer role for capsaicin (Ramos-Torres et al., 2016). In another study, capsaicin treatment inhibited nasopharyngeal carcinoma cell growth and induced autophagy, resulting in Fra-1 degradation and apoptosis (Lin et al., 2017). Interestingly, capsaicin-induced autophagy was shown to trigger the degradation of mutant p53 and reactivate wildtype p53 function, thus contributing to capsaicin-induced cytotoxicity (Garufi et al., 2016). In the present study, we found that capsaicin increased the protein levels of LC3-II and ATG5, and LC3-II fluorescence staining revealed that autophagosomes were formed in capsaicin-treated cells. In contrast, the level of p62 was only slightly increased upon capsaicin treatment. Co-treatment of capsaicin with chloroquine increased the level of LC3-II compared to that seen in cells treated with capsaicin alone. The expression level of p62 was not affected by combination treatment of cells with capsaicin plus chloroquine or the ULK1 inhibitor. The generation of ATG5-ATG12 and LC3-II are two important steps in the process of autophagosome formation. p62 is an UBA domain-containing protein that, along with its cargo protein, is incorporated into and degraded in autolysosomes. Based on our data, we hypothesized that capsaicin might contribute to both initiating autophagy and impairing the late stage of autophagy in oral squamous carcinoma cells. Our data reveal that capsaicin-induced autophagy is independent of apoptotic signaling, suggesting that capsaicin enhances autophagy to slow the cell proliferation rate via ER stress in oral squamous carcinoma cells.

Necroptosis is a subtype of necrosis that represents a regulated version of the necrotic cell death pathway. Unlike apoptosis, necroptosis is a caspase-independent death program. Recent studies have shown that there is substantial interplay between apoptosis and necroptosis. Although the precise mechanism is still unclear, it is currently thought that necroptotic cell death requires caspase inhibition and the kinase activity of receptor-interacting serine/threonine protein kinases 1 and 3 (RIPK1 and RIPK3). When necroptosis is induced, for example TNF-α stimulation or caspase 8 inhibition, RIP1 is up-regulated and binds to RIPK3, causing oligomerization and autophosphorylation of RIPK3 at Ser227. Phosphorylated RIPK3 recruits and then phosphorylates MLKL at Thr357 and Ser358 to induce a conformation change and expose the MLKL N-terminal domain, thus promoting the translocation of MLKL to the membrane. This membrane translocation of MLKL is essential for cell lysis in the necroptotic process. Degterev et al., who originally coined the term “necroptosis,” showed that this process is characterized by a necrotic cell-death morphology and activation of autophagy (Degterev et al., 2005). Further, in addition to TNF-α, several stress, such as chemotherapeutic agents, inflammatory factors, DNA damage, kinase inhibitors, natural products, or immune modulators can trigger necroptosis. Notably, the necroptosis induced by these agents is often accompanied by autophagy, which may be responsible for suppressing apoptosis and biasing cells toward necroptosis (Degterev et al., 2005; He et al., 2014). Based on our present, we propose for the first time a novel mechanism whereby capsaicin co-treatment promotes a switch from apoptosis to necroptosis when tumor cells are exposed to chemotherapeutic agents, as evidenced by dramatic down-regulation of caspase 3 and PARP and up-regulation of phospho-MLKL. Future work is needed to examine the underlying molecular mechanisms through which capsaicin induces necroptosis in the combination treatment.

Combination therapy represents a promising therapeutic strategy to overcome toxicity by reducing the effective dose of an anticancer drug. It can be particularly effective to combine chemotherapeutic drugs with different mechanisms; this may improve the therapeutic effect, decrease side effects by reducing the required dose of each drug, and help prevent multidrug resistance. The simultaneous treatment of an anticancer drug with certain phytochemicals hold promise to augment the efficacy of cancer chemotherapy. Phytochemicals have been shown to effectively potentiate the anticancer effects of conventional anticancer drugs, such as 5-FU, cisplatin, docetaxel, and doxorubicin. The molecular mechanisms and antitumor potency of phytochemicals applied in combination with anticancer drugs have been widely discussed, and have been suggested to act through antioxidant activity, anti-inflammation, enhanced apoptosis, inhibited proliferation, modulated signaling pathways, and/or altered gene expression (Tan and Norhaizan, 2019; Calcabrini et al., 2020). Capsaicin, which is a phytochemical with anticancer and chemosensitizing abilities, is considered to be safe, tolerable, and nontoxic, and can exhibit synergistic inhibitory effects with anticancer drugs against human cancers, such as osteosarcoma, colorectal adenocarcinoma, hepatocellular carcinoma, pancreatic cancer, and small lung cancer (Hong et al., 2015; Friedman et al., 2017; Vendrely et al., 2017; Dai et al., 2018; Li et al., 2018; Wang et al., 2018). Based on these accumulated results, we speculated that it could be possible to use capsaicin in combination therapy. If this strategy proves fruitful, it could be used to establish individualized therapy, especially for tumors in which apoptotic cell death cannot be induced by chemotherapeutic reagents alone. Indeed, our present results indicate that capsaicin has the potential to sensitize oral carcinoma cells to chemotherapeutic-induced cytotoxicity.

Oral squamous cell carcinoma is the most common oral cancer, representing more than 90% of the histologically classified oral tumors in men. The mainstream treatment for oral squamous cell carcinoma is the combined therapeutic strategy of surgery followed by adjuvant radiotherapy with or without concomitant use of 5-FU, cisplatin, and docetaxel. Our present study revealed that oral squamous carcinoma cells are relatively resistant to capsaicin, which did not induce significant apoptosis. Instead, we uncovered a novel mechanism whereby capsaicin induces ER stress and autophagy to down-regulate ribophorin II, which impairs P-glycoprotein functions to sensitize oral squamous carcinoma cells to anticancer drugs. Moreover, we found that the co-application of capsaicin with any of four anticancer agents promotes necroptosis instead of apoptosis to inhibit the cell viability of oral squamous carcinoma cells. Our results provide evidence supporting the idea that the co-application of capsaicin and anticancer drugs is a synergistically relevant approach for the treatment of cancers, and show for the first time that capsaicin co-treatment drastically increases anticancer agent-mediated cytotoxicity by promoting a switch from apoptosis to necroptosis.

## Data Availability

The original contributions presented in the study are included in the article/Supplementary Material, further inquiries can be directed to the corresponding author.
