# Supplementary figures and images for: Capsaicin Potentiates Anticancer Drug Efficacy Through Autophagy-Mediated Ribophorin II Downregulation and Necroptosis in Oral Squamous Cell Carcinoma Cells
Source: Front Pharmacol. 2021 Aug 27;12:676813. doi: 10.3389/fphar.2021.676813 (PMC8429935; doi:10.3389/fphar.2021.676813)

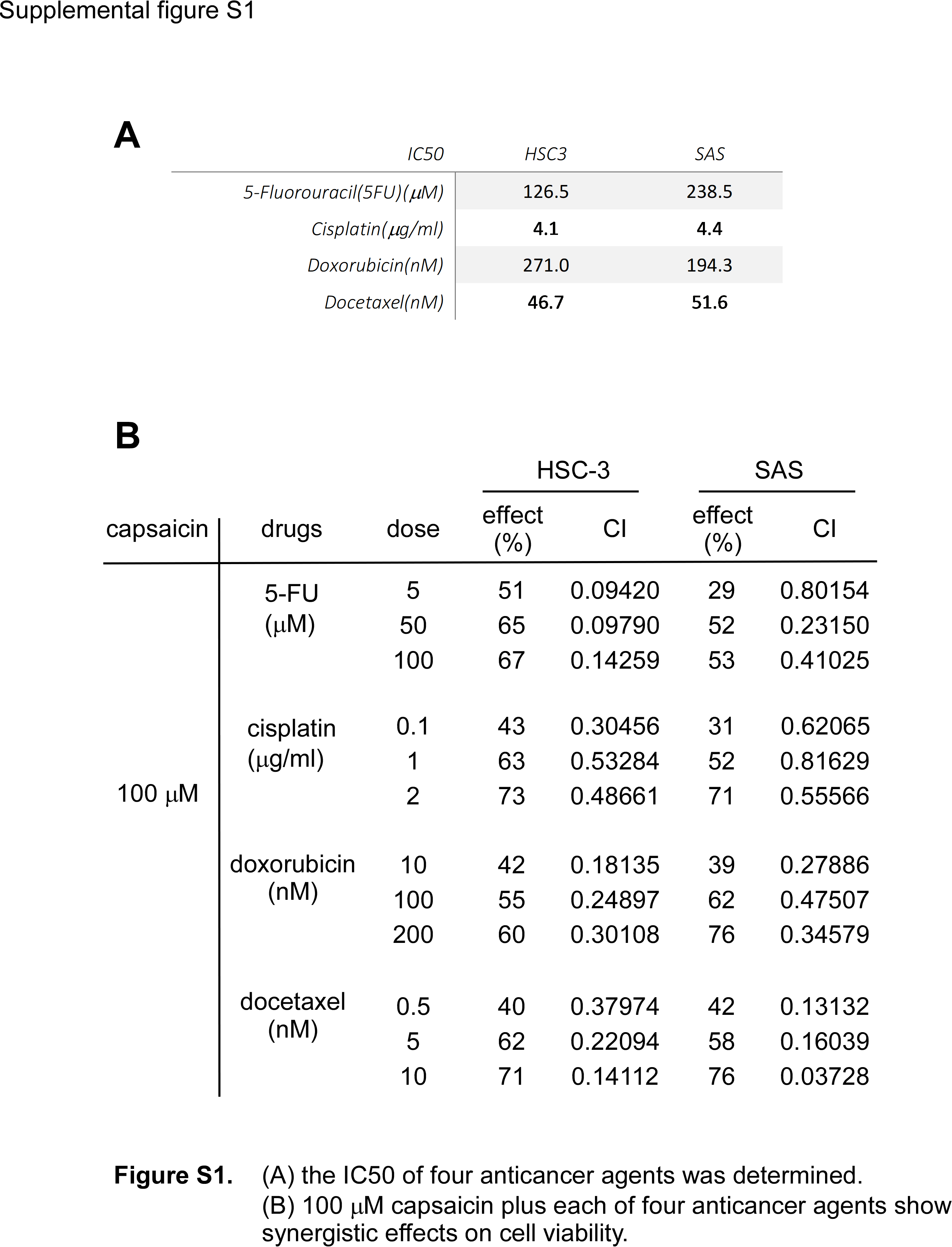

Supplement: Supplementary file 1 [file Image1.TIF]
